# Application of single-point prostate biopsy in elderly patients with highly suspected prostate cancer

**DOI:** 10.3389/fonc.2022.983805

**Published:** 2022-10-14

**Authors:** Yang Luan, Qin Xiao, Xue-fei Ding, Liang-yong Zhu, Yue-xing Han, Hao-peng Chen, Tian-bao Huang, Sheng-ming Lu

**Affiliations:** Clinical Medical College, Yangzhou University, Yangzhou, China

**Keywords:** prostate cancer, biopsy, quickly, pathology, diagnosis

## Abstract

**Objective:**

To explore the feasibility of single-point prostate biopsy in elderly patients with highly suspected prostate cancer.

**Methods:**

Forty-three patients with a prostate imaging reporting and data system score (PI-RADS) of 5, age ≥ 80 years and/or PSA ≥ 100 ng/ml and/or Eastern Cooperative Oncology Group score ≥ 2 were enrolled in our hospital from March 2020 to June 2022. Targeted surgery of these patients was performed using only precise local anesthesia in the biopsy area. The biopsy tissues were examined by intraoperative frozen section examination (IFSE). If the result of IFSE was negative, traditional systematic biopsy and further routine pathological examination were performed. The positive rate of biopsy, operation time, complications and pain score were recorded.

**Results:**

The positive rate of prostate biopsy was 94.7%. The results of IFSE in two patients were negative, and the routine pathological results of further systematic biopsy of those patients were also negative. The visual analog scale and visual numeric scale were 2 (2-4) and 3 (2-3), respectively, during the biopsy procedure. The mean time of operation was 8.5 ± 2.1 min from the beginning of anesthesia to the end of biopsy. It took 35.3 ± 18.7 minutes to obtain the pathological report of IFSE. The incidences of complication hematuria and urinary retention were 10.5% and 2.6%, respectively.

**Conclusion:**

For elderly patients with highly suspected prostate cancer, single-point prostate biopsy can be used to quickly and safely obtain pathological results.

## Background

With the continuous improvement in quality of life, diagnostic technology and treatment technology, the incidence of prostate cancer in China is also increasing year by year (1). In addition, as aging persons are coming to represent an increasing proportion of the population, prostate cancer now ranks first in the incidence of male genitourinary tumors among men over 70 years of age (2). Because of the lack of specific symptoms in the early stage of prostate cancer and its insidious onset, approximately 40% of prostate cancer patients already have advanced stage cancer when they are diagnosed (3). These patients have a poor prognosis and typically receive conservative treatment that may include endocrine therapy, chemotherapy, or immunotherapy (4–6). However, regardless of which treatment method is chosen, pathological diagnosis of prostate cancer is needed. At present, transperineal template-guided prostate biopsy (TTPB) is one of the methods most commonly used in the diagnosis of prostate cancer (7, 8). However, because prostate biopsies must penetrate the skin and subcutaneous layers and require a long time to perform, effective anesthesia is a prerequisite for success (9). Moreover, with the increase in the number of cores obtained, complications and pain also increase (10). Therefore, for elderly patients in poor general health who have clinically highly suspected advanced prostate cancer, and only for the purpose of obtaining a pathological diagnosis, this study designed a set of simple anesthesia and biopsy procedures that can be used to quickly obtain accurate pathology results without increasing the pain experienced by the patient. In this procedure, which we named “single-point prostate biopsy” (SPPB), only local anesthesia is applied to the target biopsy area; 1-2 cores are then obtained from the target area, followed by intraoperative frozen section examination (IFSE).

## Methods

### Clinical data

The clinical data of patients who underwent SPPB in our hospital from July 2020 to June 2022 were collected. The study inclusion criteria were as follows: multiparametric magnetic resonance imaging (mpMRI) showed that the prostate imaging reporting and data system (PI-RADS) score was 5; the patient was ≥ 80 years of age; and the patient had a PSA level ≥ 100 ng/ml and/or an Eastern Cooperative Oncology Group (ECOG) score ≥ 2. The exclusion criteria were as follows: presence of serious cardiovascular or cerebrovascular disease or abnormal coagulation function or the presence of severe urinary tract infection. This study was approved by the Ethics Committee of our hospital, and all enrolled patients signed informed consent forms.

### Biopsy procedure

Preparation before biopsy involved the use of a brachytherapy stepper system (fixator, template, stepper) (Mick Radio-Nuclear Instruments, Mount Vernon, NY, USA), an ultrasound machine (Flex focus 1202; BK, Naerum, Denmark) equipped with a transrectal biplanar probe, and a bard biopsy gun (MC1616 and MC1820, Bard Company, USA).

Biopsy method: The patient was placed in lithotomy position. Combined with mpMRI images, the projection range of suspicious lesions in the perineal skin was observed under the guidance of transrectal ultrasonography (TRUS). In an area 0.5 cm larger than this range, infiltration anesthesia of the perineal skin was performed using 10 ml of 1% lidocaine. Infiltration anesthesia was then performed on the prostate. The syringe was positioned so that it entered at the 1, 5, 7 and 11 o’clock positions within the area over which suspicious lesions projected in the perineal skin, and 8 ml of 1% lidocaine was injected near the prostate. After 5 min, mpMRI/TRUS cognitive fusion-guided prostate biopsy was performed.

MC1616 was used to obtain 1-2 cores from the suspected lesions, and the biopsy tissues were examined by IFSE. The patient was restored to the supine position while waiting for the IFSE pathological results. If the IFSE result was positive, the procedure was terminated. If it was negative, the lithotomy position was resumed, and a traditional systematic biopsy was performed. The biopsy tissue was examined by routine pathology.

### Pain and complication assessment

A visual analog scale (VAS; 0, none; 10, intolerable pain) was used to evaluate pain, and a visual numeric scale (VNS; 0, terrible; 4, perfect) was used to quantify the patient’s satisfaction with the procedure (11, 12). VAS-1 and VNS-1 were determined during the biopsy procedure, and VAS-2 and VNS-2 were determined 30 min after the procedure.

The patients completed self-administered questionnaires in which they reported whether they experienced complications such as hematuria, urinary retention, infection, hemospermia, or vasovagal reaction.

### Statistical methods

SPSS statistical software (version 19.0; IBM Corp, Armonk, NY, USA) was used for data processing. Kolmogorov-smirnov was performed to test whether the data were normally distributed. If normally distributed, datawere described as the mean ± SD (age, PSA concentration, prostate volume, operating time and time of pathology), otherwise were displayed as the median (interquartile range) (ECOG score and pain scores); enumeration data are expressed as percentages.

## Results

**Figure 1:**
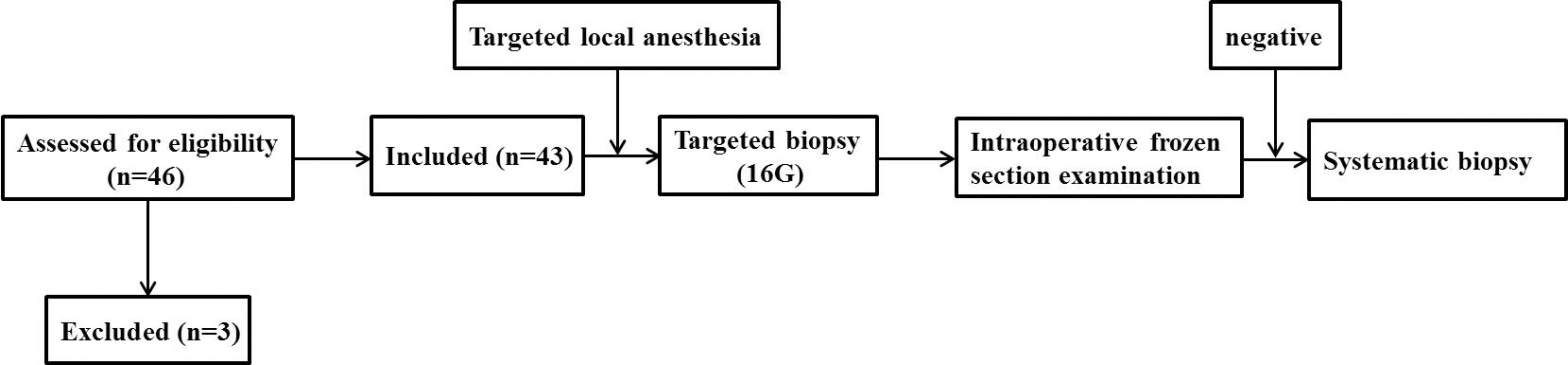
The flow diagram of the study.

A total of 43 patients who underwent single-point prostate biopsy at the Clinical Medical College of Yangzhou University were enrolled in this study (**Figure 1**). The average age, average PSA concentration and average prostate volume of the participants were 84.1± 2.5 years, 60.9 ± 21.7 μg/L and 39.3± 16.2 ml, respectively. The median ECOG score was 2 (2, 3). Forty-one of the 43 patients were diagnosed with prostate cancer, yielding a positive rate of 95.3%. The proportions of Gleason scores 6- 10 were 4.9% (2/41), 22.0% (9/41), 34.1% (14/41), 26.8% (11/41) and 12.2% (5/41) respectively. The patients’ characteristics are described in **Table 1**. Bone scans revealed bone metastases in 61.0% (25/41) of patients. Finally, 70.7% (29/41) of the patients received androgen deprivation therapy or complete hormonal therapy, and 29.3% (12/41) received treatment including radiotherapy and surgery, etc. The IFSE results of two patients were negative, and the routine pathological results of the further systematic biopsies of those patients were also negative. The age, PSA, PSAD of these two patients were 81years vs. 81years, 28.2 μg/L vs. 37.0 μg/L and 0.34 ng/ml^2^ vs. 0.27 ng/ml^2^ respectively.

**Table 1:** The characteristics of patients 80 or greater years of age with PIRADS 5 lesions and high suspicion for clinically significant prostate cancer.

Characteristics	values
Age(years)	84.1± 2.5
PSA(μg/L)	
0-20	11.6% (5/43)
20-50	32.6% (14/43)
50-100	30.2% (13/43)
>100	25.6% (11/43)
PSA density (ng/ml^2^)	0.73± 0.21
Prostate volume(ml)	39.3± 16.2
ECOG score	
2	74.4% (32/43)
3	23.3% (10/43)
4	4.7% (2/43)
Clinical stage	
cT2	65.1% (28/43)
cT3	25.6% (11/43)
cT4	9.3% (4/43)
Lymphatic metastasis	
N0	60.5% (26/43)
N1	39.5% (17/43)
Distant metastasis	
M0	76.7% (33/43)
M1	23.3% (10/43)
Gleason scores	
3 + 3	4.9% (2/41)
3 + 4	7.3% (3/41)
4 + 3	14.6% (6/41)
4 + 4	34.1% (14/41)
4 + 5	17.1 (7/41)
5 + 4	9.6% (4/41)
5 + 5	12.2% (5/41)

Regarding the scoring of pain associated with the biopsy, the VAS-1 and VNS-1 scores were 2 (2 4) and 3 (2-3), respectively. At 30 min after the procedure, the VAS-2 score was 0 (0-1), and the VNS-2 score was 3 (3-4).

The mean time from the beginning of anesthesia to the end of biopsy was 8.5 ± 2.1 min. It took 35.3 ± 18.7 min to obtain the pathological report of the IFSE.

Four patients (10.5%) developed hematuria, and the symptoms disappeared after they were instructed to drink an increased amount of water. One patient developed urinary retention after the operation; his urinary symptoms were improved after three days of indwelling catheter placement. There were no serious complications such as fever, perineal hematoma or hematospermia in any of the patients.

## Discussion

Pathological examination has always been the “gold standard” for the diagnosis of prostate cancer. It has become the consensus of major medical guidelines and clinicians and it is a policy requirement in some countries to obtain pathological diagnosis before the initiation of prostate cancer treatment, and prostate biopsy is the main means of obtaining pathology results. TTPB has the advantages of a high positive rate, high safety and the ability to extend the biopsy into the peripheral zone and other areas in which there is a high incidence of prostate cancer, as confirmed in randomized controlled studies including *in vivo* and *in vitro* experiments (13–15). Therefore, TTPB has gradually been recognized and promoted and has become one of the most common methods used in clinical practice in the diagnosis of prostate cancer. In recent years, the incidence of prostate cancer in China has been increasing, especially among patients over 70 years of age. Moreover, unlike most patients in European and American countries, most prostate cancer patients in China are already in the middle or advanced stage of the disease when they are diagnosed, and this seriously threatens the lives and the health of these patients (2, 3).

Transperineal biopsy has a low risk of infection (16). And studies have shown that the risk of severe hematuria and infection following TTPB is related to the number of cores obtained (17, 18). In addition, an increase in the number of cores will lead to additional pain, and this will increase the pain score (10). Hence, more extensive anesthesia, such as periprostatic nerve block, lumbar anesthesia, and even general anesthesia, is needed during the biopsy, and this places more stringent requirements regarding the physical condition of patients. Therefore, for elderly patients who are highly suspected of having clinically advanced prostate cancer and who cannot tolerate TTPB or conventional anesthesia due to their physical condition, a personalized prostate biopsy mode for obtaining a pathological diagnosis before treatment should be designed.

Recent improvements in MRI technology make it possible not only to scan any plane but also to obtain more accurate measurements with high tissue and spatial resolution, and this can increase the detection rate available through primary biopsy for high-risk prostate cancer (19). At the same time, the PI-RADS scoring system designed by the European Society of Urogenital Radiology can improve the diagnostic rate, allowing qualitative, localized and risk stratification of prostate cancer, increase the rate of detection of clinically significant prostate cancer, reduce the number of biopsy cores required and avoid excessive biopsy (20). In addition, with the increase in the PI-RADS score, the positive rate of biopsy for suspicious areas revealed by MRI increases accordingly (21).

Therefore, we performed targeted biopsies in patients ≥ 80 years of age with PI-RADS scores of 5 and/or PSA ≥ 100 ng/ml and/or ECOG score ≥ 2. At the same time, to avoid missed diagnosis, IFSE was conducted immediately after targeted biopsy. If the IFSE results were negative, systematic biopsy was conducted in combination with targeted biopsy. IFSE can provide pathology results in advance, and the appropriate treatment can then be initiated as early as possible according to the benign and malignant characteristics indicated by the pathology results. The burden of “cancer fear” caused by waiting for routine pathological results can be relieved. In addition, the needle used for targeted biopsy in this study was a 16G needle that was thicker in diameter than the traditional 18G needle. On the one hand, the use of a larger needle makes it easier to meet the requirements regarding the diameter and quality of the tissue needed for the IFSE. On the other hand, it indirectly achieves the same effect as increasing the number of cores and increasing the sampling volume of the biopsy and thus improves the positive rate of the biopsy procedure (22). In this study, the IFSE results after targeted biopsy showed that the positive rate of prostate biopsy was 95.3%. The IFSE results of two patients showed no sign of prostate cancer, and further systematic biopsy was performed in those patients. Patel et al (23) reported that 10-20% of patients with PIRADS 5 lesoins may not have cancer detected on targeted + systematic biopsy depending on their prior biopsy history. The two patients in this study had no previous biopsy history, which may be related to the small sample size. In addition, the results of this study show that the mean time of operation was only 8.5 ± 2.1 min from the beginning of anesthesia to the completion of the biopsy. It only took 35.3 ± 18.7 minutes to obtain the pathological report of IFSE. This not only reduces the time during which the patient experiences pain during the biopsy but also enables patients and clinicians to quickly obtain pathological results and prescribe and receive timely follow-up treatment.

In addition, to avoid risks associated with the administration of prostatic nerve block anesthesia, lumbar anesthesia, and general anesthesia, the requirement for the patient’s physical condition should be reduced, and the time during which anesthesia is administered should be reduced. In this study, only precise local anesthesia was performed on the biopsy area. The results showed that the VAS-1 and VNS-1 scores were 2 (2-4) and 3 (2-3), respectively. The patients felt almost no pain 30 minutes after biopsy. This was similar to the analgesic effect we achieved previously using targeted periprostatic nerve block anaesthesia in targeted biopsy (24). Hence, under the premise of obtaining a good analgesic effect, this method of anesthesia not only shortened the duration of anesthesia and reduced the time of anesthesia operation but also reduced the dose of anesthetic required.

This study has several limitations. First, the selection of the best biopsy location in the lesions requires further study. For patients with multiple lesions, the selection of optimal biopsy locations still requires further accumulation of techniques and experience. Although VAS and VNS are relatively objective pain-detection indicators, the assessment of pain is still based on the subjective feelings of patients and lacks objective quantitative indicators. Therefore, further studies are needed. However, because this study is a single-center study involving a small number of cases, it has certain limitations. Additional data and a multicenter, randomized controlled clinical study are needed to confirm this hypothesis.

In conclusion, in patients ≥ 80 years of age with PI-RADS scores of 5 and/or PSA ≥ 100 ng/ml and/or ECOG scores ≥ 2, SPPB can be used to quickly obtain reliable pathological results without increasing the number of cores required or the amount of pain experienced by the patient. It is a safe and effective mode of biopsy that is worthy of further promotion and application. In the future, our research group will introduce robot positioning system into the SPPB to perform more accurate targeted biopsy for patients. In addition, it has been reported patients with high suspicion of prostate cancer on prostate-specific membrane antigen positron emission tomography (PSMA- PET) and mpMRI were directly treated with a radical prostatectomy without prior biopsy (25). Therefore, our research group intends to apply PSMA- PET to elderly patients with PIRADS 5 lesions, to explore whether patients with PSMA- PET showing the possibility of prostate cancer can be directly treated with antitumor therapy without experiencing the pain and risk of operation.

## Data availability statement

The raw data supporting the conclusions of this article will be made available by the authors, without undue reservation.

## Ethics statement

The studies involving human participants were reviewed and approved by Ethics Committee of Clinical Medical College, Yangzhou University. The patients/participants provided their written informed consent to participate in this study.

## Author contributions

YL: study design and conceptualization, data acquisition, analysis and interpretation of data, and drafting the manuscript. QX: data acquisition, analysis, and interpretation of data. X-FD: study design and conceptualization, data acquisition, analysis and interpretation of data, and manuscript revision. L-YZ, Y-XH, and H-PC: data acquisition, analysis, and interpretation of data. T-BH and S-ML: data acquisition and manuscript revision. All authors contributed to the article and approved the submitted version.

## Funding

This work was supported the Yangzhou Science and Technology Plan Project (no. YZ 2019053).

## Conflict of interest

The authors declare that the research was conducted in the absence of any commercial or financial relationships that could be construed as a potential conflict of interest.

## Publisher’s note

All claims expressed in this article are solely those of the authors and do not necessarily represent those of their affiliated organizations, or those of the publisher, the editors and the reviewers. Any product that may be evaluated in this article, or claim that may be made by its manufacturer, is not guaranteed or endorsed by the publisher.

## References

[1] Chen W Sun K Zheng R Zeng H Zhang S Xia C. Cancer incidence and mortality in China. Chin J Cancer Res (2018) 30:1–12. doi: 10.21147/j.issn.1000-9604.2018.01.01 29545714 PMC5842223

[2] Fu ZT Guo XL Zhang SW Zheng RS Zeng HM Chen R. Statistical analysis of incidence and mortality of prostate cancer in China, 2015. Chin J Oncol (2020) 42(09):718–22. doi: 10.3760/cma.j.cn112152-20200313-00200 32988152

[3] Han J Yang DL Liu JH Li BH Kan Y Ma S. Family-centered psychological support helps improve illness cognition and quality of life in patients with advanced prostate cancer. Natl J Androl (2020) 505–12. doi: 10.13263/j.cnki.nja.2020.06.004 33356038

[4] Shore ND Saad F Cookson MS George DJ Saltzstein DR Tutrone R. Oral relugolix for androgen-deprivation therapy in advanced prostate cancer. N Engl J Med (2020) 382:2187–96. doi: 10.1056/NEJMoa2004325 32469183

[5] Muniyan S Rachagani S Parte S Halder S Seshacharyulu P Kshirsagar P. Sildenafil potentiates the therapeutic efficacy of docetaxel in advanced prostate cancer by stimulating NO-cGMP signaling. Clin Cancer Res (2020) 26:5720–34. doi: 10.1158/1078-0432.CCR-20-1569 PMC7642013 32847934

[6] Patel VG Oh WK. The evolving landscape of immunotherapy in advanced prostate cancer. Immunotherapy (2019) 11:903–12. doi: 10.2217/imt-2019-0019 31161846

[7] Chen Y Zhou Z Zhou Y Wu X Xiao Y Ji Z. Development and internal validation of a prediction model of prostate cancer on initial transperineal template-guided prostate biopsy. BMC Urol (2021) 21:68. doi: 10.1186/s12894-021-00840-5 33892696 PMC8063345

[8] Ding XF Luan Y Wang F Xu YZ Huang TB Guo CH. Application of modified transperineal template-guided prostate biopsy in the diagnosis of prostate cancer. Chin J Urol (2019) 040:763–7. doi: 10.3760/cma.j.issn.1000-6702.2019.10.009

[9] Udeh EI Amu OC Nnabugwu II Ozoemena O. Transperineal versus transrectal prostate biopsy: our findings in a tertiary health institution. Niger J Clin Pract (2015) 18:110–4. doi: 10.4103/1119-3077.146991 25511354

[10] Ding XF Luan Y Wang F Xu YZ Xu JN Zhou YQ. Research on the influencing factors of periprostatic nerve block anaesthesia. Chin J Urol (2018) 39:842–6. doi: 10.3760/cma.j.issn.1000-6702.2018.11.011

[11] Tufek I Akpinar H Atug F Obek C Esen HE Keskin MS. The impact of local anesthetic volume and concentration on pain during prostate biopsy: a prospective randomized trial. J Endourol (2012) 26:174–7. doi: 10.1089/end.2011.0344 22092389

[12] Doganca T Savsin A Erdogan S Altindas F Ozdemir F Ekici B. Procedural sedation and analgesia as an adjunct to periprostatic nerve block for prostate biopsy: A prospective randomized trial. J Clin Ultrasound (2015) 43:288–94. doi: 10.1002/jcu.22227 25155750

[13] Vis AN Boerma MO Ciatto S Hoedemaeker RF Schroder FH van der Kwast TH. Detection of prostate cancer: a comparative study of the diagnostic efficacy of sextant transrectal versus sextant transperineal biopsy. Urology (2000) 56:617–21. doi: 10.1016/S0090-4295(00)00681-6 11018617

[14] Mai Z Xiao Y Yan W Zhou Y Zhou Z Liang Z. Comparison of lesions detected and undetected by template-guided transperineal saturation prostate biopsy. BJU Int (2017) 121:415–20. doi: 10.1111/bju.13977 28771912

[15] Muthuveloe D Telford R Viney R Patel P. The detection and upgrade rates of prostate adenocarcinoma following transperineal template-guided prostate biopsy - a tertiary referral centre experience. Cent Eur J Urol (2016) 69:42–7. doi: 10.5173/ceju.2016.675 PMC4846721 27123325

[16] Basourakos SP Alshak MN Lewicki PJ Cheng E Tzeng M DeRosa AP. Role of prophylactic antibiotics in transperineal prostate biopsy: A systematic review and meta-analysis. Eur Urol Open Sci (2022) 37:53–63. doi: 10.1016/j.euros.2022.01.001 35243391 PMC8883190

[17] Wang F Ding XF Xu JN Xu YZ Zhou YQ Luan Y. Complications of transperineal template-guided prostate mapping biopsy. Natl Med J China (2019) 99:428–31. doi: 10.3760/cma.j.issn.0376-2491.2019.06.009 30786336

[18] Ghafoori M Velayati M Aliyari Ghasabeh M Shakiba M Alavi M. Prostate biopsy using transrectal ultrasonography; the optimal number of cores regarding cancer detection rate and complications. Iran J Radiol (2015) 12:e13257. doi: 10.5812/iranjradiol.13257 26060552 PMC4457971

[19] Zhang Q Wang W Yang R Zhang G Zhang B Li W. Free-hand transperineal targeted prostate biopsy with real-time fusion imaging of multiparametric magnetic resonance imaging and transrectal ultrasound: single-center experience in China. Int Urol Nephrol (2015) 47:727–33. doi: 10.1007/s11255-015-0957-5 25820744

[20] Weinreb JC Barentsz JO Choyke PL Cornud F Haider MA Macura KJ. PI-RADS prostate imaging - reporting and data system: 2015, Version 2. Eur Urol (2016) 69:16–40. doi: 10.1016/j.eururo.2015.08.052 26427566 PMC6467207

[21] Wang Q Zhang Q Zhang B Shi J Fu R Li DY. Free-hand transperineal multiparametric magnetic resonance imaging/transrectal ultrasound fusion-guided targeted biopsy for the diagnosis of prostate cancer: a prospective study. Chin J Urol (2018) 39:192–6. doi: 10.3760/cma.j.issn.1000-6702.2018.03.009

[22] Ding XF Luan Y Xia AL Zhu LY Xiao Q Chen J. Application of 16 G biopsy needle in transperineal template-guided prostate biopsy. Urol Int (2022) 106:909–13. doi: 10.1159/000520373 34915528

[23] Patel HD Koehne EL Shea SM Bhanji Y Gerena M Gorbonos A. Risk of prostate cancer for men with prior negative biopsies undergoing magnetic resonance imaging compared with biopsy-naive men: A prospective evaluation of the PLUM cohort. Cancer (2022) 128:75–84. doi: 10.1002/cncr.33875 34427930

[24] Ding XF Luan Y Wang F Xu YZ Guo CH Zhu LY. The application of a targeted periprostatic nerve block in transperineal template-guided prostate biopsies. Quant Imaging Med Surg (2020) 10:2125–32. doi: 10.21037/qims-20-369 PMC7547263 33139992

[25] Meissner VH Rauscher I Schwamborn K Neumann J Miller G Weber W. Radical prostatectomy without prior biopsy following multiparametric magnetic resonance imaging and prostate-specific membrane antigen positron emission tomography. Eur Urol (2022) 82:156–60. doi: 10.1016/j.eururo.2021.11.019 34887117

